# Closure of the Human TKFC Active Site: Comparison of the Apoenzyme and the Complexes Formed with Either Triokinase or FMN Cyclase Substrates

**DOI:** 10.3390/ijms20051099

**Published:** 2019-03-04

**Authors:** Joaquim Rui Rodrigues, José Carlos Cameselle, Alicia Cabezas, João Meireles Ribeiro

**Affiliations:** 1 Laboratório Associado LSRE-LCM, Escola Superior de Tecnologia e Gestão, Instituto Politécnico de Leiria, P-2411-901 Leiria, Portugal; 2 Grupo de Enzimología, Departamento de Bioquímica y Biología Molecular y Genética, Facultad de Medicina, Universidad de Extremadura, E-06006 Badajoz, Spain; camselle@unex.es (J.C.C.); acabezas@unex.es (A.C.)

**Keywords:** triokinase, dihydroxyacetone kinase, FMN cyclase, phosphoryl transfer mechanism, protein domain mobility, active-site closure, normal mode analysis, molecular dynamics simulation, essential dynamics

## Abstract

Human triokinase/flavin mononucleotide (FMN) cyclase (hTKFC) catalyzes the adenosine triphosphate (ATP)-dependent phosphorylation of D-glyceraldehyde and dihydroxyacetone (DHA), and the cyclizing splitting of flavin adenine dinucleotide (FAD). hTKFC structural models are dimers of identical subunits, each with two domains, K and L, with an L2-K1-K2-L1 arrangement. Two active sites lie between L2-K1 and K2-L1, where triose binds K and ATP binds L, although the resulting ATP-to-triose distance is too large (≈14 Å) for phosphoryl transfer. A 75-ns trajectory of molecular dynamics shows considerable, but transient, ATP-to-DHA approximations in the L2-K1 site (4.83 Å or 4.16 Å). To confirm the trend towards site closure, and its relationship to kinase activity, apo-hTKFC, hTKFC:2DHA:2ATP and hTKFC:2FAD models were submitted to normal mode analysis. The trajectory of hTKFC:2DHA:2ATP was extended up to 160 ns, and 120-ns trajectories of apo-hTKFC and hTKFC:2FAD were simulated. The three systems were comparatively analyzed for equal lengths (120 ns) following the principles of essential dynamics, and by estimating site closure by distance measurements. The full trajectory of hTKFC:2DHA:2ATP was searched for in-line orientations and short distances of DHA hydroxymethyl oxygens to ATP γ-phosphorus. Full site closure was reached only in hTKFC:2DHA:2ATP, where conformations compatible with an associative phosphoryl transfer occurred in L2-K1 for significant trajectory time fractions.

## 1. Introduction

Human triokinase/flavin mononucleotide (FMN) cyclase (hTKFC) is a bifunctional enzyme which catalyzes the adenosine triphosphate (ATP)-dependent phosphorylation of D-glyceraldehyde (GA) and dihydroxyacetone (DHA), and also the Mn^2+^-dependent splitting of flavin adenine dinucleotide (FAD) by an internal cyclizing reaction that forms adenosine monophosphate (AMP) and riboflavin 4′,5′-phosphate or cyclic FMN [1,2]. GA kinase represents the third step of fructose metabolism [3,4,5] and DHA kinase is necessary for the metabolism of exogenous DHA [6,7,8,9] and the eventually generated endogenous DHA [10]. On the other hand, the biological role of the cyclase activity is unknown [2,11,12]. hTKFC is functionally and structurally similar to *Citrobacter* sp. DHA kinase, which displays similar activities, including GA kinase and FMN cyclase [6,10,12,13]. The *Citrobacter* DHA kinase is also of biotechnological interest due to its efficient use for in situ production of dihydroxyacetone phosphate (DHAP) in systems developed for C–C bond formation catalyzed by DHAP-dependent aldolases [14,15].

**Figure 1:**
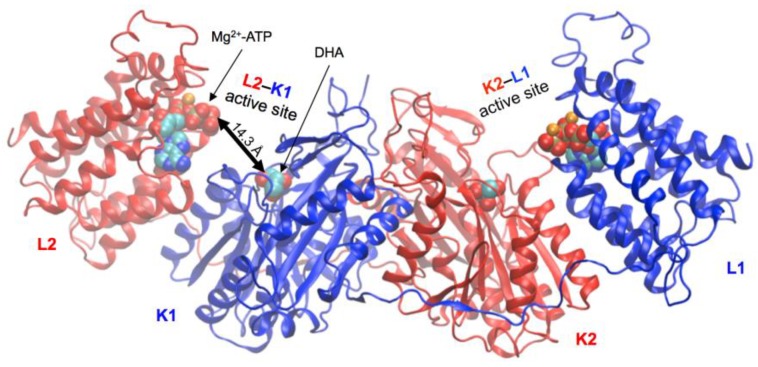
Homology model of human triokinase/flavin mononucleotide (FMN) cyclase (hTKFC) in complex with dihydroxyacetone (DHA), adenosine triphosphate (ATP) and Mg^2+^ (hTKFC:2DHA:2ATP). The ATP-to-DHA distance is indicated in the L2-K1 site. The description is in the text. The figure was drawn according to the coordinates reported elsewhere [2].

*Citrobacter* DHA kinase has a known crystal structure with a unique fold [16] that defines Group 9 in the classification of kinases [17,18]. This enzyme, and the hTKFC model constructed by homology [2], show up a homodimer (in agreement with size exclusion chromatography data for hTKFC [2]), each subunit with two domains, K and L, linked by a long spacer (Figure 1). The intertwined subunits form an elongated L2-K1-K2-L1 arrangement. Two kinase active sites lie between domains L2-K1 and K2-L1, where triose binds covalently to histidine residues of the K domains and ATP binds to the L domains in complex with two Mg^2+^ ions.

In the crystal structure of *Citrobacter* DHA kinase and in the homology model of hTKFC (Figure 1), the ATP-to-triose distance is about 14 Å, too large for phosphoryl transfer [2,16]. In fact, from the structural data nothing can be inferred about the mechanism, and it has been suggested that bringing ATP near the triose requires mobility of the protein domains in solution [10,16]. In previous work, to study the flexibility of dimeric hTKFC starting with its active sites in an open conformation, we ran a 75-ns simulation of the molecular dynamics of the structural model of hTKFC containing the kinase substrates DHA (actually a trihydroxyprop-2-yl radical covalently bound to His^221^) and ATP (hTKFC:2DHA:2ATP; Figure 1). The trajectory of this simulation shows the protein acquiring a less open conformation in one of the active sites, with significant decrease of the ATP-to-DHA distance, which in the 34–37-ns and 47–55-ns intervals reaches frequently distances around 5 Å, and include very transient minima of 4.83 Å at 34.860 ns and 4.16 Å at 54.622 ns. After this, the ATP-to-DHA distance increases up to around 10 Å [2].

The main purpose of this study was to look for further confirmation of the trend towards active-site closure and to analyze whether substrate binding could influence protein domain mobility. In particular, we wondered if results could be obtained to support the movements required for a direct phosphoryl transfer from ATP to the triose as suggested [10,16]. To address these questions, different studies have been performed. On the one hand, the principles of normal mode analysis were applied to the hTKFC:2DHA:2ATP complex typical of the phosphoryl transfer reaction, to the hTKFC:2FAD typical of FMN cyclase activity, and to the apo form of hTKFC. On the other hand, the earlier 75-ns trajectory of hTKFC:2DHA:2ATP was extended up to 160 ns, and studied in parallel with new dynamic simulations of hTKFC:2FAD and apo-hTKFC of 120 ns. The three trajectories were comparatively analyzed for equal lengths (120 ns each) following the principles of essential dynamics and by estimating the degree of active-site closure through distance measurements between proper reference points in the K and L domains. Finally, in search for optimal conformations for the in-line attack of DHA over ATP (phosphoryl transfer), the two active sites of the hTKFC:2DHA:2ATP dimer were analyzed along the full 160-ns trajectory, measuring the distances from each DHA hydroxyl oxygen to the ATP γ-phosphorus, and the angles formed by these oxygen-phosphorus pairs with the scissible P–O linkage of ATP. 

## 2. Results

### 2.1. Normal-Mode Analysis of hTKFC Models Confirms the Trend of K Domains to Approach L Domains

**Figure 2:**
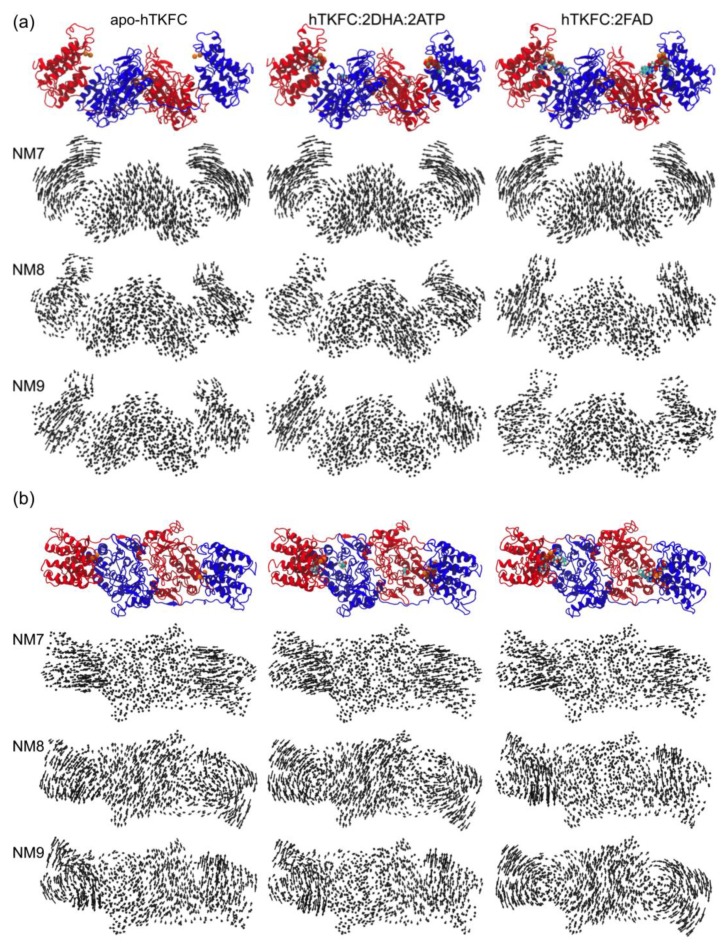
Porcupine representation of the first three non-trivial normal modes NM7–NM9 for the apo form of hTKFC and its complexes with kinase or FMN cyclase substrates. The arrows indicate the direction and extent of the motion for each Cα atom. Other atoms were omitted for simplicity. (**a**) “Lateral” view, similar to Figure 1, (**b**) “top” view. The first line of each panel shows the energy-minimized structures used for the normal mode analysis. An animated representation can be seen in Supplemental movie 1. The correspondence with the three universal modes of bilobate structures is indicated in Figure 3.

**Figure 3:**
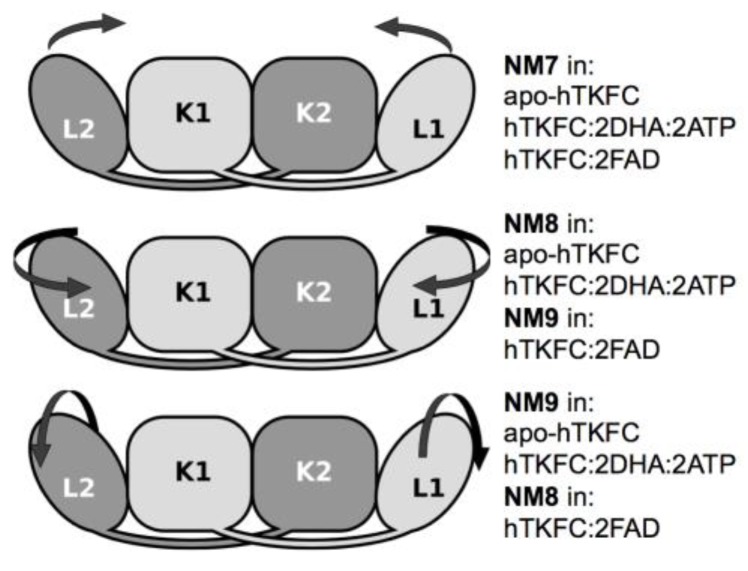
Schematic interpretation of the major movements contained in non-trivial normal modes shown in Figure 2. These movements can be compared to the three universal modes [22] defined for bilobate structures: (upper) hinge-bending, (middle) twisting, (lower) wobbling. Additional descriptions of the NM7–NM9 modes can be found in the text. In some cases, the normal modes of L1 and L2 differ slightly (see the text).

The analysis of normal modes is a major theoretical method to predict collective movements in proteins [19,20,21]. Three systems were analyzed by this approach: apo-hTKFC, hTKFC:2DHA:2ATP, and hTKFC:2FAD. After the necessary thorough energy minimization, the conformations of these systems differed from the initial ones with Cα root-mean-square deviation (RMSD) values of 1.11 Å, 1.50 Å and 1.16 Å, respectively. Hessian matrices calculated from the minimized conformations were used to calculate eigenvectors (normal modes) and their associated eigenvalues. In this type of analysis, the first six normal modes (NM1–NM6) are considered trivial and disregarded. Therefore, the non-trivial normal modes NM7–NM9 obtained for the three systems are represented in Figure 2 as porcupine plots. Supplemental movie 1 shows the domain movements corresponding to NM7–NM9 for the three systems, and a schematic interpretation is made in Figure 3. 

**Figure 4:**
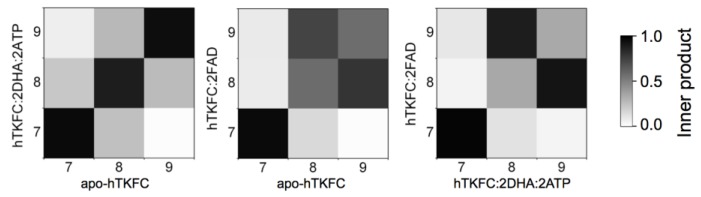
Comparison of normal modes NM7–NM9 for the three systems studied. A set of conformations was generated for each system and mode, and the information about their Cα atoms was extracted such that the same number of atoms was considered in each case. The resulting trajectories were submitted to principal component analysis from where eigenvectors and eigenvalues were derived. Inner products were calculated for each comparison and are displayed in a grey scale: from 1.0, identity, to 0.0, orthogonality.

To compare the normal modes of the systems studied, besides the possibility of direct visual comparison in Figure 2 and Supplemental movie 1, quantitative comparisons can be made in terms of eigenvector inner products which would equal unity if vectors are identical or zero if orthogonal. The results of the pairwise comparison among the three systems studied are shown in Figure 4. 

The first non-trivial modes (i.e., NM7) were very similar in the three systems. The L1(L2) domain tilted towards the K2(K1) domain in parallel to the main axis of the protein dimer that runs approximately through the mass centers of the L2-K1-K2-L1 domains (Supplemental movie 1). The similitude of the NM7 modes of the three systems was confirmed by the corresponding inner products being near to unity (Figure 4). This mode is similar to the so-called hinge-bending mode defined for bilobate structures formed by two globular domains linked by a flexible hinge [22], although in hTKFC, L2-K1 (K2-L1) are not directly linked. The NM7 mode may be part of a functional movement in the case of the hTKFC:2DHA:2ATP system, as its prolongation would bring about closer contacts between the L and K domains, and therefore the needed approximation of ATP to DHA for the kinase reaction. 

The second non-trivial modes of apo-hTKFC and hTKFC:2DHA:2ATP (i.e., NM8) were similar to each other, and to the third non-trivial mode (NM9) of hTKFC:2FAD (Figure 4). They consisted of a partial twist of the L domain around a vertical axis located between the K and L domains (similar to the twisting mode defined for bilobate structures [22]), combined with a slight tilt perpendicular to the movement in NM7 (Supplemental movie 1). The prolongation of the NM8 mode of hTKFC:2DHA:2ATP, like NM7, would also bring about some approximation between ATP and DHA. 

The third non-trivial modes of hTKFC and hTKFC:2DHA:2ATP (i.e., NM9) were similar to each other, and to the second non-trivial mode (NM8) of hTKFC:2FAD (Figure 4). They consisted of a wobbling of the L domain about a horizontal axis approximately parallel to the main axis of the protein dimer (similar to the wobbling mode defined for bilobate structures in [22]) without bringing about any obvious approximation between L and K domains (Supplemental movie 1). Therefore, its functional character in hTKFC:2DHA:2ATP is less clear, but cannot be discarded when combined with the other modes. 

The analysis of normal modes showed a clear trend of the open conformation of hTKFC to progress towards a less open conformation. However, this was little dependent on the presence of ligands. In fact, hinge-bending, which is the kind of movement that most clearly evokes the closure of the active site, is the one with the lowest oscillation frequency for the three systems (3.79–4.46 cm^−1^) with barely noticeable differences among them, the differences consisting of slight deviations from the movement parallel to the main axis of the protein dimer described above. 

On the other hand, the twisting and wobbling movements of the three systems showed more accentuated differences, regardless of the rather narrow range of the frequencies associated to these modes in the three systems: NM8 4.74–5.51 cm^−1^; NM9 5.35–5.80 cm^−1^. Despite the symmetry of dimeric hTKFC, these movements were not exactly equivalent for both L domains. The differences are not easy to grasp from the observation of Supplemental movie 1. The twisting modes of the L2 domain were very similar in the three systems, but less so in L1, where the twist of apo-hTKFC (and, to a lesser extent, hTKFC:2DHA:2ATP) was mixed with a noticeable wobbling originating from the top of the domain. The wobbling modes of the L2 domains of apo-hTKFC and hTKFC:2DHA:2ATP differed from that of hTKFC:2FAD in their axes, while those of the L1 domains differed in that the latter system, but not the two former ones, was mixed with a slight hinge-bending movement and a horizontal axis of rotation closer to the top of the domain. 

In summary, although the normal modes of the three systems studied differed only in subtle form, it is possible that these small differences of modes can explain the larger differences that were seen in molecular dynamics simulations (see below).

### 2.2. Comparison of hTKFC-2DHA-2ATP, hTKFC-2FAD and apo-hTKFC Molecular Dynamics Trajectories

**Figure 5:**
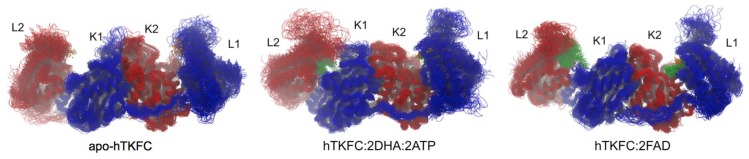
Molecular dynamics of hTKFC and its complexes with kinase or cyclase substrates. The figure shows the superimposition of 60 snapshots extracted from 120-ns trajectories at 2-ns intervals and aligned with the initial conformations.

The normal mode analysis of the systems apo-hTKFC, hTKFC:2DHA:2ATP and hTKFC:2FAD indicated that there could be subtle differences in their harmonic movements perhaps due to the absence or presence of different ligands. Thus, considering that the previously published study of dynamics involved only hTKFC:2DHA:2ATP [2], it was interesting to compare molecular dynamics trajectories of the different systems. The comparison was performed along 120-ns simulations. In the case of hTKFC:2DHA:2ATP, the previously published 75-ns simulation was prolonged up to 120 ns, whereas the 120-ns trajectories of apo-hTKFC and hTKFC:2FAD were simulated de novo. The three 120-ns trajectories are summarized in Figure 5 by superimposing conformations extracted at regular intervals of 2 ns and aligned with the initial structures. The three simulations showed the overall stability of the complexes, which conserved their dimeric character, the organization of the L and K domains, and their elongated disposition. However, the three systems showed significant fluctuations that affected more the L domains than the centrally located K domains and, in general, affected more the regions devoid of secondary structure than those with it. Upon visual comparison of the three sets of superimposed conformations, the most remarkable differential feature was the closure of one of the active sites of hTKFC:2DHA:2ATP by a marked L2-to-K1 approximation not observed in apo-hTKFC or hTKFC:2FAD (Figure 5).

**Figure 6:**
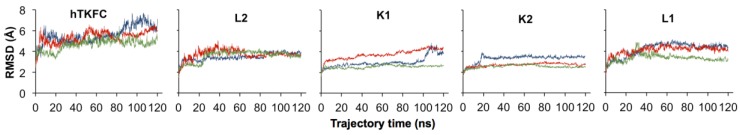
Root-mean-square deviations (RMSD) of Cα atoms during molecular dynamics simulation of hTKFC and its complexes with kinase or cyclase substrates. Distances were calculated relative to their initial positions in the structural models prepared. The different panels show, as indicated, the RMSD values calculated for the full protein or for each protein domain separately. The colored traces correspond to (blue) apo-hTKFC, (red) hTKFC:2DHA:2ATP and (green) hTFKC:2FAD. Each system was first submitted to an energy-minimizing and equilibration process, upon which the trajectory production phase was started. At time zero the three systems differed from the initial ones with Cα RMSD values of 2.57 Å, 2.39 Å and 2.56 Å, respectively.

The divergence of the three systems with respect to the respective initial structures, after their alignment, was quantitated in terms of RMSD of the Cα positions (Figure 6). The increasing deviations converged towards a plateau with RMSD about 5 Å when all the Cα atoms of hTKFC were considered. RMSD values calculated separately for each L or K domain were smaller than those for the full proteins, an indication of the occurrence of movements of one domain with respect to the other. In general, the K domains showed higher conformational stability than the L ones. Upon comparison of the three systems, there was no consistent effect of the presence of hTKFC ligands DHA, ATP or FAD. However, it must be stated that RMSD values do not give information on the vectorial aspects of the movements. As major RMSD variations are seen in the first 10 or 20 ns, the subsequent analysis of the molecular dynamics trajectory was restricted to the 20–120 ns time period.

**Figure 7:**
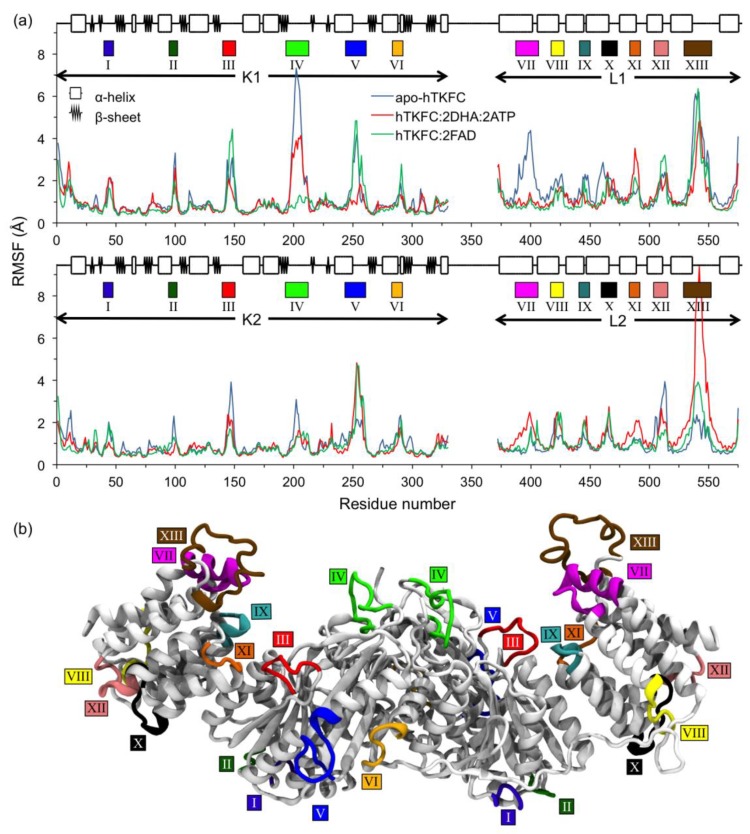
Distribution of mobility in the peptide chains of the hTKFC systems. (**a**) Root-mean square fluctuations (RMSF) of Cα atom positions relative to their mean positions during the 20–120-ns molecular dynamics simulations. Data for subunits 1 and 2 are in separate panels. (**b**) Location of the regions with higher fluctuation in the structural model of apo-hTKFC before the dynamics. The colored rectangles below the secondary structure schemes in (**a**) and those in (**b**) mark these regions in the two peptide chains. Only one region VI is visible in part (**b**).

Figure 7a shows the distribution of Cα mobility along the two peptide chains of the three systems. In this figure, mobility is presented in terms of fluctuation relative to the mean position of each atom (RMSF). Thirteen high mobility regions were observed in each peptide chain, six of them located in the K (I–VI) and seven in the L domains (VII–XIII). They are colored in the structure of apo-hTKFC shown in Figure 7b and marked by similarly colored rectangles in both parts of Figure 7. In the K domains, most of the mobility was located within protein loops. This was so in mobile regions I–IV, while V and VI included significant portions of α-helices. In the L domains, the seven mobile regions corresponded each with a protein loop but included in every case significant portions of α-helices. In no case did high mobility affect β strands (present only in the K domains). With a few exceptions, the mobile regions behaved similarly in both peptide chains. Most conspicuous was the large difference in mobility of the two regions VII and, to a lesser extent, the two regions IX. In L1, region VII was highly mobile in apo-hTKFC but showed little fluctuation in hTKFC:2DHA:2ATP and hTKFC:2FAD. Similar but less marked behavior was observed in L1 region IX. Region VII of L2, compared to L1, showed much lesser fluctuation in the case of apo-hTKFC, and higher mobility in hTKFC:2DHA:2ATP. L2 region IX did not show differences among the three systems. The differences observed in the mobility of regions L1 VII and L1 IX, between apo-hTKFC and the systems containing bound substrates, correlate with the proximity of bound ATP or FAD to these parts of L1 (see Figure 1). In this regard, the different responses of L1 and L2 to bound substrates (Figure 7a) are intriguing, and may be related to the different degree of closure of L2-K1 and K2-L1 sites during the molecular dynamics of hTKFC:2DHA:2ATP (Figure 5).

**Figure 8:**
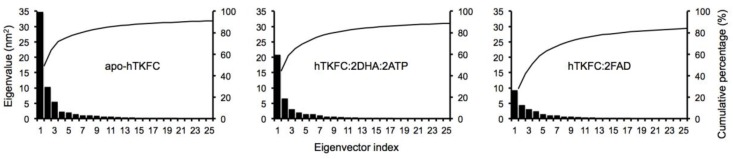
Essential dynamics of hTKFC and its complexes with kinase or cyclase substrates. The first 25 eigenvectors or principal components are shown in decreasing order of eigenvalue. The bars represent the eigenvalues. The lines represent the cumulative percentages of eigenvalues.

**Figure 9:**
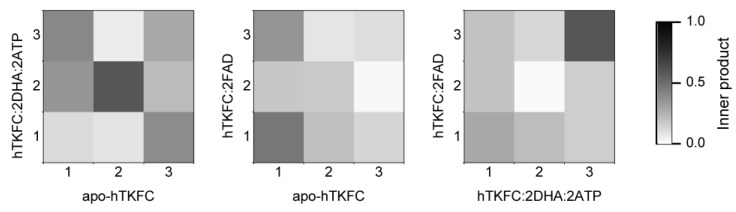
Comparison of principal components 1–3 for the systems studied. Inner products for each comparison are displayed in a grey scale: from 1.0, identity, to 0.0, orthogonality.

**Figure 10:**
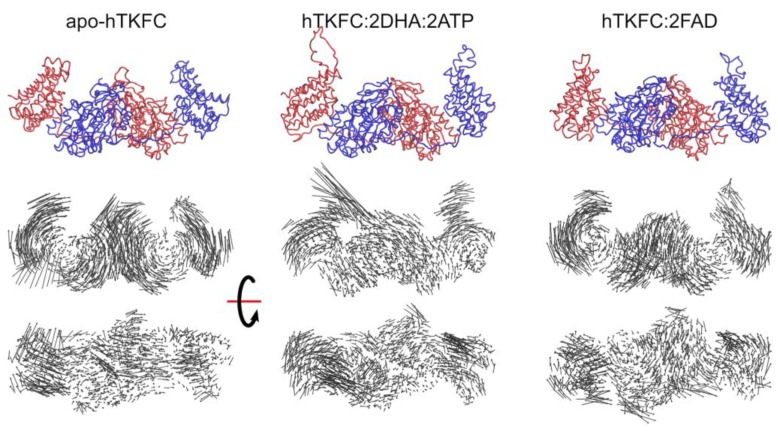
Porcupine representation of principal component 1 for the systems studied. The first line shows the more open of the two extreme conformations defined by the first principal component. The central and lower lines show, respectively, “lateral” and “top” views of the arrow sets indicating the direction and extent of the motion for each Cα atom. An animated representation can be seen in Supplemental movie 2.

Principal component analysis was applied to reveal the most important movements in the trajectories of apo-hTKFC, hTKFC:2DHA:2ATP, and hTKFC:2FAD, i.e., their essential dynamics, and also as an alternative method to normal mode analysis [23]. The magnitudes of the first 25 principal components or eigenvectors are shown in Figure 8. In all cases, the first component was predominant over the others, although the difference was less marked in the hTKFC:2FAD system. The first 3 eigenvectors explain 68% of the variance of the apo-hTKFC and hTKFC:2DHA:2ATP systems, and 52% of hTKFC:2FAD complex. Of course, similar variances do not mean similarity in terms of direction. In this concern, the results of the pairwise comparison of the first 3 eigenvectors of the three systems studied are presented in Figure 9, which shows that the principal components of hTKFC:2DHA:2ATP and hTKFC:2FAD differed from those of apo-hTKFC, but also that those of the two substrate-containing systems differed from each other. Static representations of the first eigenvector of each system are shown in Figure 10 in the form of porcupine plots, while an animated representation can be seen in Supplemental movie 2. Figure 10 shows that an important contribution to principal component 1 was made by the movement of the L2 domain loop labeled as XIII in Figure 7. It is worth noting that this region is disordered in the crystallized *Citrobacter* DHA kinase [16] used as a template to model hTKFC [2] (see also Section 4.1). Actually, the large mobility of loop XIII is consistent with the fact that these amino acids are not visible in the electron density map of the *Citrobacter* protein. As displayed in Figure 10 and in the Supplemental movie 2, this loop seems to play an important role in the closure of the L2-K1 site.

### 2.3. Active-Site Closure in the hTKFC-2DHA-2ATP Complex: Conformations for In-Line Nucleophilic Attack and Phosphoryl Transfer from ATP to DHA

The major reason to undertake this molecular dynamics study was to investigate the closure of hTKFC active site as the possible way for ATP and triose, which in the initial structure bind quite apart from each other (Figure 1), to reach an optimal conformation for an in-line phosphoryl transfer. For a hypothetical attack operating through an associative mechanism [24], such conformation should display both the proper distance (e.g., <3.5 Å) between the γ-phosphorus atom of ATP and one of the primary hydroxyl oxygens of the trihydroxyprop-2-yl radical covalently bound to His^221^, and an adequate angle (e.g., >155°) formed by this oxygen-phosphorus pair with the scissible P–O linkage of ATP. 

**Figure 11:**
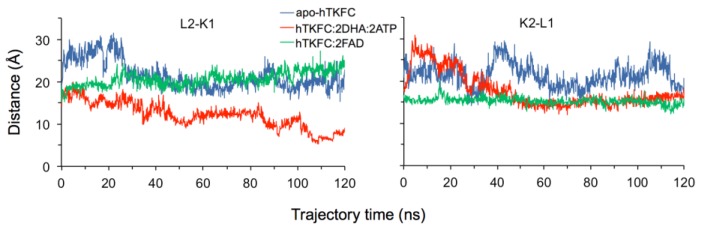
Estimation of the closure of the active sites of hTKFC and its complexes with kinase or cyclase substrates during the simulated trajectories. Distances were measured between Mg2 in the L domain and the Nε2 of His^221^ in the K domain of the same site, i.e., L2-K1 or K2-L1.

One important aspect of this question was to evaluate the influence of substrates bound to hTKFC on the trend towards active-site closure. As stated above, the direct visual inspection of the trajectories summarized in Figure 5 makes clear the marked closure of the L2-K1 site in the presence of DHA and ATP, not in the other systems. To make this a quantitative comparison, distances between marker atoms in the L domain and the K domain of the same site (i.e., L2-K1 or K2-L1), irrespectively of the presence or absence of ligands, were taken from the 120-ns trajectories of the three systems. The marker atoms were chosen by their involvement in the hTKFC:2DHA:2ATP complex in the binding of ATP or DHA. The atoms selected were Mg2, which is coordinated with the L domain and interacts with the γ-phosphoryl group of ATP when present, and the Nε2 atom of His^221^, to which triose is covalently bound when present. The shortening of the Mg2···Nε2 distance was used as an indication of active-site closure, and its evolution during the 120-ns trajectories in the two active sites of the three systems is shown in Figure 11. In agreement with the observations of Figure 5, active-site closure was clearly favored by the presence of bound ATP and DHA, although only in the L2-K1 site, not in the K2-L1 one. 

Once it was established that ATP and DHA bound to hTKFC favored at least the closure of one of the active sites, we scanned the 160-ns trajectory of hTKFC:2DHA:2ATP for the two parameters which delimit the possibility of in-line nucleophilic attack of DHA (the trihydroxyprop-2-yl radical covalently bound to His^221^) over the γ-phosphorus of ATP: O···P distance and O···P–O angle. Since the two hydroxyl groups of DHA (and of the trihydroxyprop-2-yl radical) are equivalent, two O···P pairs were considered in each active site.

**Figure 12:**
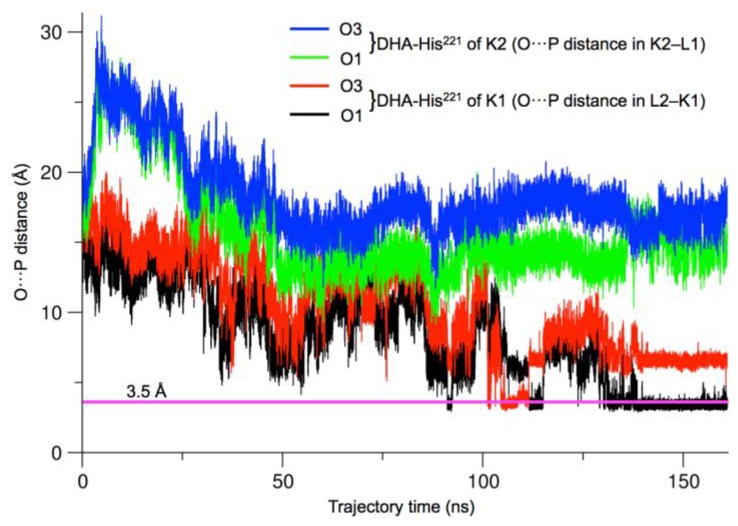
Evolution of the DHA-to-ATP distance in the hTKFC:2DHA:2ATP trajectory. Distances were measured in the full 160-ns trajectory between each of the hydroxyl oxygens of DHA (the trihydroxyprop-2-yl radical covalently bound to His^221^) and the γ-phosphorus of ATP in the same site (i.e., L2-K1 and K2-L1). The pink line marks the 3.5 Å distance. The first 75-ns of this trajectory have been reported in earlier work [2].

**Figure 13:**
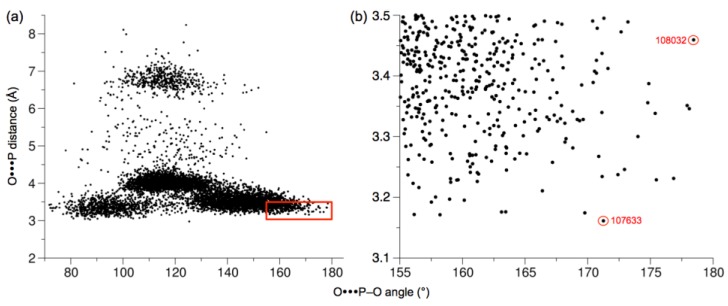
Combination of O···P distances and O···P–O angles in the L2-K1 site during the hTKFC:2DHA:2ATP trajectory in the 105–112-ns time range. The points correspond to snapshots extracted at 1-ps intervals and indicate O···P distance from the DHA O3 oxygen to the P atom, and the corresponding O···P–O angle. (**a**) Data for the 7000 snapshots. (**b**) Magnification of the red rectangle of panel a, including 395 snapshots. The conformations marked in red in the close-up are identified by its time in picoseconds (shown in Figure 14).

**Figure 14:**
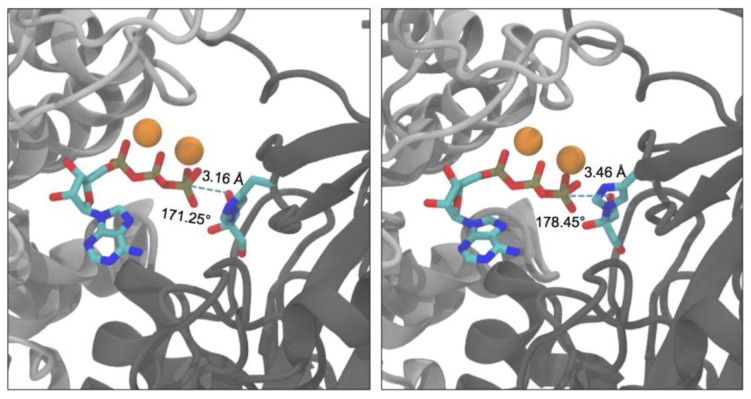
Selected near attack conformations of hTKFC:2DHA:2ATP detected during the molecular dynamics trajectory in the L2–K1 site. Light gray, L2 domain; dark gray, K1 domain. The images correspond to snapshots 107633 (left) and 108032 (right-hand side).

Figure 12 shows the scan of O···P distance in the two active sites. Distances <3.5 Å were found in the L2-K1 site (not in K2-L1) for significant time fractions of the trajectory, either with one or the other DHA hydroxyl oxygen atom. This occurred, with DHA O1, at times 91–92 ns, 111–115 ns and 130–160 ns, and with DHA O3, at 105–112 ns. Therefore, the O···P–O angle was scanned only in the L2-K1 site from 90 ns to 160 ns, to search for the coincidence of adequate distances (<3.5 Å) with good angles (>155°). These coincidences were found only for DHA O3 within the 105–112 ns range. The data for the 7000 snapshots of this range are presented in Figure 13a. The lower-right part of the distribution corresponds to the snapshots displaying the shorter O···P distances and the larger O···P–O angles. The data for the 395 snapshots with O···P distances <3.5 Å and O···P–O angles >155° are shown in Figure 13b. Any of them is a plausible conformation for an in-line phosphoryl transfer. Two of these snapshots are shown as examples in Figure 14, one selected because it displays the shortest O···P distance (3.16 Å), the other because it displays the largest O···P–O angle (178.45°). 

## 3. Discussion

hTKFC catalyzes the ATP-dependent phosphorylation of DHA and GA, and the splitting of FAD to form cyclic FMN. In previous work a detailed enzymatic study of the protein was undertaken. This included probing the role of several amino acids by mutagenesis and the individual expression of the K and L protein domains [2]. The main objective of the current paper was to get further insights on the role of protein domain mobility into the mechanism of the DHA kinase activity of hTKFC. 

Two enzymatic systems are known that phosphorylate DHA [10]. On the one hand, in eukaryotes and some bacteria, including *Citrobacter*, this reaction is catalyzed by enzymes which require ATP as cosubstrate, and are commonly known as ATP-dependent DHA kinases. It is not known whether all these enzymes are also able to convert FAD in cyclic FMN, but this is true at least for the *Citrobacter* enzyme [12]. The crystal structure of *Citrobacter* DHA kinase is available [16] and it was used by us as template to model hTKFC [2]. Both the bacterial enzyme and the model of the human one are homodimeric proteins formed by chains (1 and 2) having each two domains, K and L, connected by an extended, 20-amino acids linker. ATP, in complex with two Mg^2+^ ions, binds to domain L, whereas DHA binds covalently to domain-K His^221^ (hTKFC; His^220^ in *Citrobacter*). As described (see Figure 1), these proteins display an elongated intertwined structure L2-K1-K2-L1, with two active sites per dimer, located in the interfaces between, respectively, K1 and L2, and K2 and L1 domains. It may be assumed that putative ATP-dependent DHA kinases sharing high homology with hTKFC, will have a similar general structure. On the other hand, in most bacteria the DHA kinase system is composed of three randomly interacting proteins DhaK, DhaL and DhaM, which have been enzymatically and structurally studied in e.g., *Escherichia coli* [25,26,27] and *Lactococcus lactis* [28]. Rather than using ATP as a cosubstrate, this system depends on ADP tightly bound to DhaL as a prosthetic group, which in each enzyme cycle is alternatively phosphorylated to ATP by DhaM, a phosphoprotein of the phosphoenolpyruvate:sugar phosphotransferase system (PTS), and dephosphorylated when the phosphoryl group of ATP is transferred to DHA covalently bound to DhaK [29]. However, important similarities exist between the ATP-dependent and the PTS-dependent kinases. First, the DhaK and DhaL subunits of PTS-dependent DHA kinases are homologous to the K and L domains of the ATP-dependent DHA kinases, both in sequence and function. Second, the *E. coli* DhaK-DhaL complex crystallizes as a heterotetramer formed by two DhaK and two DhaL monomers, in which the overall disposition DhaL-DhaK-DhaK-DhaL resembles the L2-K1-K2-L1 structure of the hTKFC homodimer. Third, in both groups of enzymes, DHA is bound to a His residue by a hemiaminal linkage. 

The crystal structure of the *E. coli* DhaK–DhaL complex contains ADP and DHA [27]. When ADP is replaced by ATP (i.e., the phosphorylated form of the cofactor), the γ-phosphoryl atom of ATP appears located 3.9 Å away from the 1(3)-OH group of DHA, and the orientation of DhaL relative to DhaK is consistent with a direct transfer of the γ-phosphoryl moiety from ATP to a terminal hydroxyl group of DHA. Taking into account the structures obtained, a general three-step mechanism has been proposed for PTS-dependent DHA kinases: formation of the hemiaminal linkage, direct phosphoryl transfer from ATP to DHA, and release of DHAP [27]. Recently, a theoretical study of this mechanism has been performed using quantum mechanics/molecular mechanics methods [30].

Contrary to what is seen in the *E. coli* enzyme, in the crystal structure of the *Citrobacter* DHA kinase the ATP molecule and the DHA moiety lie too far apart (≈14 Å) for an in-line phosphoryl transfer to take place, and attempts to crystallize alternative more closed conformations have been unsuccessful. It has been suggested that K- and L-domains, connected just by a long spacer, could move with respect to each other, and that domain mobility would be required for kinase activity [10,16]. The relative mobility of both domains has been actually shown by us in a previous work with the homology model of hTKFC, where, in the course of a 75-ns trajectory of molecular dynamics, transient ATP-to-DHA approximations are seen in the L2-K1 site, displaying apparent near-attack conformations that, however, last very shortly [2].

In the current work, we have followed different approaches that confirm the trend towards closure of at least one of the hTKFC active sites. On the one hand, normal mode analysis indicated that the first non-trivial mode of the protein displayed precisely this trend, although this was independent of the presence or absence of substrates in the protein complex (Figure 2, NM7 mode). On the other hand, when the simulation of molecular dynamics was extended beyond the original 75 ns, the L2-K1 site resumed its closure movements and reached closer ATP-to-DHA approximations, including O···P distances <3.5 Å measured between the entering hydroxyl oxygen and the attacked phosphorus atom, which lasted for several nanoseconds (Figure 12). These approximations to <3.5 Å distances included many snapshots in which the O···P–O angle was near 180°, pointing to an almost perfect in-line attack conformation (Figure 13). The molecular dynamics of apo-hTKFC and hTKFC:2FAD showed a much less marked trend towards active-site closure (Figure 11). In fact, such movement is unlikely to have any significance for the FMN cyclase activity of hTKFC, as it has been shown that the isolated L domain is almost as efficient cyclase as the dimeric protein [2]. 

Regarding the fact that during the 160-ns molecular dynamics trajectory only one of the two active sites reaches a closed state, several arguments may be made. One possibility is that both centers could not close simultaneously. However, from our kinetic studies it seems that the enzyme follows a standard hyperbolic kinetics, with no signs of substrate cooperativity, either positive or negative [2]. So, it seems that the behavior of one active site would not affect the other. Furthermore, normal mode analysis suggested that both sites could actually move towards a closed conformation (Figure 2). More likely, it could be that a longer molecular dynamics trajectory could be needed to obtain a fully closed enzyme. Actually, from our former kinetic studies [2] it is known that the DHA kinase activity of hTKFC has a *k*_cat_ of 5 s^−1^, i.e., one catalytic event each 200 ms. Therefore, the 160 ns simulation is far from covering the time length required for a full catalytic event.

From the results discussed above, it may be assumed that, despite the large difference of relative positioning of ATP and DHA in PTS-dependent and ATP-dependent DHA kinases, inferred from structural data, the phosphoryl group is transferred directly from ATP to enzyme-bound DHA in both enzyme kinds, without any enzyme intermediary phosphorylation step. The general chemical mechanisms of phosphoryl transfer have been reviewed by several authors [24,31,32,33,34]. In principle, these transfer reactions may be described as following either a stepwise (associative or dissociative) or a concerted mechanism.

A stepwise, fully associative addition-elimination mechanism proceeds via a bipyramidal intermediate in which the phosphorus atom of a phosphorane intermediate is simultaneously bound to the entering and leaving oxygen. At a near-attack conformation, the entering oxygen should be at a distance of ≈3.3 Å of the phosphorus, i.e., the van der Waals sum of both atoms (P, 1.9 Å; O, 1.4 Å). Remarkably, in our simulation of the molecular dynamics of hTKFC:2DHA:2ATP, starting from a distance ≈14 Å, conformations easily compatible with a fully associative mechanism were reached (Figure 13b and Figure 14).

A stepwise, fully dissociative elimination-addition mechanism proceeds via the separation of the phosphoryl group from the donor to form a metaphosphate intermediate that then reacts with the acceptor. At the intermediate step, when the metaphosphate intermediate exists, there could be contact between the P atom and both oxygens, but not bonding. At this time, both P–O distances would be ≥3.3 Å. If one assumes that, once the attack conformation is reached, the relative positions of the entering and leaving oxygens do not change significantly, in the near-attack conformations of Figure 13b and Figure 14 there would be no room for a fully dissociative reaction.

The two kinds of stepwise mechanisms may better represent two extreme situations, while many enzymes would follow an alternative one-step concerted mechanism, in which breaking and forming of the phosphorus bonds with the leaving and entering oxygen atoms occurs more or less simultaneously. As these processes do not necessarily occur synchronously, a concerted reaction can proceed through different types of transition states, ranging from more loose (or dissociative-like) to more tight (or associative-like) ones. The near-attack conformations of Figure 13b and Figure 14 are compatible also with this kind of mechanism.

In summary, as during the molecular dynamics trajectory, very short distances were reached between the ATP γ-phosphoryl group and the DHA moiety (Figure 13b and Figure 14), it seems unlikely that the phosphoryl transfer between both substrates could follow a fully dissociative reaction. So, the results presented here for the DHA kinase activity of hTKFC would more likely point to either an associative mechanism or a concerted mechanism with a (more or less) tight transition state.

## 4. Materials and Methods

### 4.1. Structural Models of hTKFC

The coordinates for hTKFC:2DHA:2ATP were those of a homology model constructed with Modeller [2] using two crystal structures of *Citrobacter* sp DHA kinase proteins as templates, one in apo form and the other containing bound ATP-Mg and DHA covalently bound to histidine [16]. The model of apo-hTKFC was obtained by manual removal of ATP and DHA (actually the trihydroxyprop-2-yl radical covalently bound to His^221^) from hTKFC:2DHA:2ATP also as described [2].

The structural model of hTKFC:2FAD was obtained by modification of the published model of hTKFC:FAD which contains only one FAD bound to the L domain of subunit 1 [2]. Starting from this hTKFC:FAD model, the second FAD molecule was added to the L domain of subunit 2 using a transformation matrix, calculated with the VMD program version 1.9.1, that allowed to align subunit 1 with subunit 2. Slight atomic overlaps appearing in the binding of FAD to the second site were removed by energy minimization of the complex.

The use of a homology model to study molecular dynamics adds a degree of uncertainty to the simulations as compared with the study of molecular dynamics based on crystal structures. This could be seen as an important limitation of the study. However, the models seem quite reliable, as hTKFC shares with the templates a 40% identity evenly distributed along the 575 amino acid sequence, while model and template display an overall RMSD of 0.7 Å. Actually, 40% identity is well within the safe homology modeling zone for protein alignments of this length [35,36]. The major concern deals with the completeness of the templates themselves. In the published structures of the *Citrobacter* DHA kinase, the amino acids aligning with residues 541–548 of hTKFC are missing, indicating that they are rather disordered [16]. Therefore, to be able to run the molecular dynamics simulation those residues were modeled de novo [2]. This part of the protein corresponds to the loop labeled XIII in Figure 7.

The composition of the three systems used for molecular simulations is summarized in Table 1.

### 4.2. Normal-Mode Analyses

These simulations were run with GROMACS 4.0.7 [37] using the AMBER03 force field [38]. The topologies and parameters of *N*^τ^-(1,2,3-trihydroxyprop-2-yl)-l–histidine, ATP and FAD are available under access codes F-86 and F-91 in the R.E.DD.B. database [39]. For the normal mode analyses, the three structural models of hTKFC obtained as described above were used in vacuo: apo-hTKFC, hTKFC:2DHA:2ATP, and hTKFC:2FAD. Prior to the analyses, the models were optimized with the GROMACS MD engine mdrun by running 5000 energy-minimization iterations by the steepest descent method, followed by 10,000–15,000 iterations with the low memory Broyden–Fletcher–Goldfarb–Shanno algorithm, such that the maximal force in any atom of the minimized structures was below 10^−6^ kJ mol^−1^ nm^−1^. For the energy minimizations, van der Waals and electrostatic interactions up to a distance of 10 Å were taken into account, although above 8 Å a corrective function was applied to make null the resulting energies of interactions at 10 Å. The atomic coordinates of the minimized conformations were used to calculate, also using mdrun, their hessian matrices (**H**), which are composed by the elements of Equation (1)

(1) $$H_{ij}=\frac{\partial^{2}V}{\partial x_{i}\partial x_{j}}$$

x_i_ and x_j_ being the cartesian coordinates of i and j atoms, and V the potential energy for their interaction. Eigenvectors and eigenvalues were determined from the hessian matrices with the program g_nmeig according to Equation (2)

(2) $$\Lambda =\mathrm{diag}\left(\lambda_{1}\cdots \lambda_{3N}\right)=RM^{-1/2}HM^{-1/2}R^{T}$$

where **Λ** is a diagonal matrix with 3N eigenvalues λ_i_, **R** is a matrix with eigenvectors in columns, **R^T^** is the transpose of **R**, and **M** is a matrix containing the masses of the N atoms considered (Table 1). The eigenvectors or normal modes are 3N-dimensional unitary vectors that describe the direction of collective atom movement, whereas the eigenvalues λ_i_ yield the frequencies of the normal modes through Equation (3)

(3) $$\upsilon_{i}=\frac{\sqrt{\lambda_{i}}}{2\pi }$$

After ordering the normal modes by increasing frequencies and disregarding the six trivial ones (NM1–NM6), which display the lowest frequencies, the next three modes, i.e., NM7, NM8 and NM9, were analyzed. Starting with the minimized conformations and the eigenvectors, the program g_nmtraj was used to generate trajectories that allowed visualization of the harmonic oscillations of each mode. Porcupine plots, drawn with the Normal Mode Wizard tool of VMD [40], were used as static representations of the normal modes. 

Quantitative comparisons between the normal modes of molecular systems with different numbers of atoms needs their reduction to sets of atoms shared in common. Therefore, to allow for the comparison of the normal modes of apo-hTKFC, hTKFC:2DHA:2ATP, and hTKFC:2FAD, the following approach was used. For each system and normal mode, a set of 5000 conformations was generated at 300 K with the g_nmens program included in GROMACS. From these conformations, the Cα, C, O and N atoms of the protein main chain were extracted, what gave the same atom number (4600) in every case. Principal component analyses of these sets of conformations, run as described in Section 4.4, allowed the eigenvalues and eigenvectors characteristic of each normal mode to be obtained. For the conformations for a single normal mode, only the first eigenvalue is significant while the rest are near null. To evaluate the similitude between normal modes of different systems, the inner products of their protein main chain eigenvectors were calculated, which adopted values between 1, indicative of identity, and 0, indicative of orthogonality.

### 4.3. Molecular Dynamics

The trajectories of molecular dynamics of apo-hTKFC, hTKFC:2DHA:2ATP, and hTKFC:2FAD were simulated in the presence of explicit solvent (water and NaCl) using GROMACS 4.0.4 [37] and the AMBER03 force field [38] supplemented like for the normal mode analysis. The conditions of the simulations, including the preliminary energy-minimizing process and equilibration steps, were as previously reported for a 75-ns trajectory recorded for hTKFC:2DHA:2ATP [2]. This was prolonged up to 160 ns under the same conditions, which were used also to record the 120-ns trajectories of apo-hTKFC and hTKFC:2FAD.

### 4.4. Essential Dynamics

Principal component analysis was applied to the last 100 ns of the 120-ns trajectories recorded for the molecular dynamics of apo-hTKFC, hTKFC:2DHA:2ATP, and hTKFC:2FAD. To simplify the calculations, the procedure was applied only to the 1150 Cα atoms of each dimeric system (i.e., 575 per subunit). The g_covar program, included in GROMACS, was used to align 100,001 conformations of each system with the initial conformation to remove translation and rotation motions, thus generating **x**(t) trajectories (that can be viewed as matrices of size 3 × 1150 coordinates × 100,001 conformations), from which symmetric covariance matrices (**C**) of size 3450 × 3450 were calculated. The elements of **C** are given by Equation (4)

C_ij_ = 〈(x_i_ − 〈x_i_〉) (x_j_ − 〈x_j_〉)〉 (4)

where 〈〉 represent time-averaged values. The same program was used to diagonalize **C** and to obtain **Λ** and **R** matrices

(5) $$\Lambda =\mathrm{diag}\left(\lambda_{1}\cdots \lambda_{3450}\right)=R\;C\;R^{T}$$

where **Λ** is a diagonal matrix with 3450 eigenvalues λ_i_ in decreasing order, **R** is a 3450 x 3450 matrix with eigenvectors in its columns, and **R^T^** is the transpose of **R**. The i column of **R** is the eigenvector associated to the λ_i_ eigenvalue of **Λ**. Each eigenvector describes a collective movement of atoms and the associated eigenvalue is the variance of its fluctuations and represents the magnitude of the collective movement. The analysis of eigenvectors and eigenvalues was made with the g_anaeig program, also part of GROMACS.

## 5. Conclusions

Computational evidence supports a marked trend of hTKFC:2DHA:2ATP, i.e., the complex typical of the DHA kinase activity of hTKFC, towards active-site closure in one of the two sites displayed by the dimeric enzyme.

Normal mode analysis indicates a clear trend of both active sites towards closure in the first non-trivial normal mode. However, by this approach, no major, but only subtle differences are observed with respect to the normal modes of hTKFC:2FAD, the complex typical of the FMN cyclase activity of hTKFC, or of apo-hTKFC, the substrate-free enzyme.

By contrast, molecular dynamics simulations run up to 120 ns with the three hTKFC systems indicate that one of the hTKFC:2DHA:2ATP active sites undergoes a full closure, with a very strong approximation of ATP to DHA, from about 14 Å in the initial state to below 3.5 Å. No such degree of active-site closure is observed in hTKFC:2FAD or apo-hTKFC, indicating that this marked movement is specific to the enzyme complex ready to catalyze phosphoryl transfer. Principal component analysis of the three trajectories supports also this specificity.

In the course of the molecular dynamics trajectory of hTKFC:2DHA:2ATP, after 100 ns, many conformations are reached with the geometry needed for a direct in-line attack of one of the primary hydroxyl oxygens of DHA over the γ-phosphate of ATP. This includes O···P distances below 3.5 Å (some around 3.3 Å, the theoretical contact distance between oxygen and phosphorus) and O···P–O angles larger than 155° (some very near to 180°). According to theory, this is compatible with an associative mechanism or with a concerted mechanism with a rather tight transition state.

Despite the reported inability to find crystal structures of the *Citrobacter* DHA kinase (an ortholog of hTKFC) showing ATP-to-DHA distances below 14 Å, there is no need to invoke an indirect phosphoryl transfer to explain the kinase activity of these enzymes. Such activity can be accounted for by the marked flexibility of their dimeric structure with active sites formed between domains of different subunits.

## Figures and Tables

**Table 1:** Composition of the systems submitted to normal-mode analysis and molecular dynamics.

	apo-hTKFC	hTKFC:2DHA:2ATP	hTKFC:2FAD
Components used for both procedures			
hTKFC chains	2	2	2
Mg^2+^	4	4	4
DHA	0	2	0
ATP	0	2	0
FAD	0	0	2
Components used only for molecular dynamics			
Na^+^	84	84	84
Cl^−^	94	86	90
H_2_O	87,364	87,570	87,304
Total number of atoms			
In normal mode analysis	16,814	16,924	16,982
In molecular dynamics	279,084	279,804	279,068

## Supplementary Materials

Supplementary materials can be found at https://www.mdpi.com/1422-0067/20/5/1099/s1.

## Abbreviations

ATP	Adenosine 5′-triphosphate
DHA	Dihydroxyacetone
DHAP	Dihydroxyacetone phosphate
FAD	Flavin adenine dinucleotide
FMN	Flavin mononucleotide
GA	d-Glyceraldehyde
hTKFC	Human triokinase/FMN cyclase
RMSD	Root-mean-square deviation
PTS	Phosphoenolpyruvate:sugar phosphotransferase system
